# Notes and News

**Published:** 1889-05-11

**Authors:** 


### Notes and News.

Collected and Communicated.

As the governors of the Halifax Hospital have determined to erect a new hospital, it is hoped they will show commendable judgment in the selection of plans by placing the work in the hands of one of the few architects who have devoted their attention to hospital construction. They would be well advised to go to Hastings and inspect the new hospital there in the first instance, as they will thus see what it is possible to do in many directions on sound principles at a reasonable cost.

On Thursday, May 2, a delightful concert was given at Prince's Hall in aid of the East London Hospital at Shadwell. Madame Osborn Williams, Miss Agnes Zimmerman, Miss Henrietta Cowen, and a number of other eminent artistes assisted to carry out a programme which was as varied as it was pleasing. The ever good-natured Duchess of Teck gave her patronage, and the Marquis de Lenville took the opportunity to recite some of his own verses. A substantial sum has been handed over to the hospital.

The annual report of the Royal Mineral Water Hospital at Bath states that the number of applications for admission has been 1,521, for the year. On the 1st May last 160 patients were in the house, and during the year just closed 1,133 have been admitted, and 1,137 have been discharged. The committee point with pleasure to the fact that not a single death has occurred in the house during the past year. The daily average in the house has been 149, and the average stay of each patient 48 days. Through the year applicants for admission have had to wait a considerable time for vacancies, and at the present time 84 men and 60 women are waiting. In consequence of this it has been decided to increase the accommodation by providing five additional beds for females, and the enlargement of the hospital has been under discussion.

The Ilkley Hospital and Convalescent Home issues its report for last year. The number of patients admitted, including renewals, was 1,027, as compared with 968 last year, being the largest number recorded in the history of the hospital. Since the erection of the hospital in 1861, the total number of admissions and renewals amounted to 18,758. The receipts for the year, including the balance from 1887 of £148 14s. 5d., amounted to £1,888 19s. 10d., and the expenditure, including the sum of £130 placed on fixed deposit, to £1,633 11s. 10d., leaving a balance in favour of the current account of £255 8s. The working expenses of the year were £1,503 11s. 10d., and the cost per patient was £1 9s. 3d., as against £1 8s. in 1887. The Rev. Canon Jackson gave an interesting sketch of the rise and progress of this admirable institution.

A grand bazaar has been held at Bournemouth in aid of the Royal Victoria Hospital. Lady Wimborne opened the bazaar, which was held in the new hospital buildings, and the crowd of visitors was so great that the receipts should amount to a sum of four figures. Lady Wimborne made an excellent little speech, and set a good example by purchasing liberally of the many beautiful wares offered at the different stalls. The ladies of Bournemouth are to be congratulated on the enthusiastic manner in which they have worked for the hospital.

At the festival dinner of the Samaritan Free Hospital last week contributions amounting to £5,000 were announced, including £1,000 from the ever charitable Mr. John S. Morgan. A great many ladies were present, and amongst the gentlemen were Dr. Bantoch, Dr. Routh, and other well-known members of the medical profession. The committee have decided to erect at once a portion of the new building, which will cost about £12,000, and provide sixty-three beds for patients. Statistics show that a very large number of cases have been successfully treated, and that the benefits afforded to women suffering from special complaints have recently increased. Excellent work has, it is stated, also been done on behalf of children in the limited space which the committee can devote to children's wards.

A sad story comes to us from Durham. A short time since two distinct diseases broke out in the village of New Herrington. One took the form of typhoid, and the other pleuro-pneumonia, and people of all ages were attacked by one or the other of them. So rapidly did the epidemic spread, that it was deemed necessary to report to the Local Government Board. Drs. Pollard and Page, on behalf of that body, have inspected the locality and held an enquiry touching the outbreak, the result of which will be known in a few days. Upwards of 200 cases are reported in New Herrington alone, and the outbreak has spread to the adjacent villages of Philadelphia, Newbottle, Hetton, New Lambton, and Burnmoor, at all of which places fatal cases are reported. A complete army of medical men, trained nurses, and clergymen are in constant attendance upon the afflicted populace.

On May 1st Mr. S. C. Lister laid the foundation-stone of the Children's Hospital, Bradford, and marked the occasion by giving the munificent donation of £5,000 towards the project. The whole of the proceedings were successfully carried through, and the luncheon which followed the ceremony was lively and the speeches interesting. The sisters of All Saints first started the Children's Hospital in 1883, and it was found to fill such a much-needed want that it was shortly made a public affair and greatly enlarged. Still, a new building with all modern improvements was needed, so the plans of Messrs. H. and E. Martin were accepted and are now being carried out. The buildings occupy a site of about one acre at the corner of St. Mary's-road and Welbury-drive, with a frontage to the latter of 100 yards. The principal elevation, facing south, is to this road. The style of architecture adopted is a simple character, of the type prevalent during the Queen Anne period, and appears well suited for hospital work. The design is striking and somewhat novel, and when the circular wards are completed it will take a position as a place of interest in Bradford. The most important hospitals in this country and on the Continent were studied by the architects before finally settling their plans, in order that they should embrace all the latest improvements in hospital construction, and it is believed that the working of the hospital will be economically and successfully carried on.

The following vacancies are announced this week, the amount of the salary in each case being given after the name of the institution :

**Resident Medical Officers.**

Hospital for Women, Soho. Two Clinical Assistants.
Derbyshire Infirmary. House Surgeon.—£100, board, &c.
Ipswich Hospital. Assistant House Surgeon. — £20, board, &c.
Torbay Hospital. Junior House Surgeon and Dispenser.—£80, board, &c.
North Staffordshire Infirmary. House Surgeon.—£100, board, &c.
Dudley Guest Hospital. Medical Officer.—£100, board, &c.
Risbridge Union. Medical Officer.—£70.

**Non-Resident Medical Officers.**

University of Durham. Lecturer on Anatomy.—£300.
Victoria University. Lecturer on Diseases of Women and Children.

---

Lord Suffield laid the foundation-stone of Gorleston Cottage Hospital on May 2nd. It will be remembered that a cottage hospital was opened about fourteen months ago at Gorleston, it being determined to celebrate the Queen's Jubilee by establishing an institution of that sort, and so great have been its benefits that it was determined to build a new and larger hospital, the site for which was given by Mr. H. Harvey George. The new building is to cost £1,000. Mr. Dudley Arnott is the honorary architect.

---

The annual meeting of the governors of the London Homœopathic Hospital was held on the last day of April, Lord Ebury presiding. The report stated that the year had been one of much activity in every department. The recent largely increased number of patients treated had been maintained; the demand for the services of the trained nurses of the hospital had been fairly sustained. The income had increased, and the expenditure had declined. The convalescent home had been opened, and had already done a good and increasing work, and the entire outlook is one of hopefulness and encouragement. Sorrow was expressed at the death of the Duchess of Cambridge, who was a warm friend of the institution during the thirty-nine years of its existence, and the Duchess of Teck was asked to take the place of the deceased lady. They had lost a warm friend of the hospital by the death of Lord Alfred Paget, one of its vice-presidents from its commencement. Another loss was the removal by death of Sir James Alexander, for seven years a member of the board, and who left £500 to the convalescent home.

---

Some interesting facts about a certain Dr. Snipe, surgeon, reach us from Geneva, where they have been gravely read by M. Fatio before the Physical Society. On one occasion M. Fatio killed a snipe which had on the chest a large dressing composed of down from other parts of the body, and securely fixed to the body by coagulated blood. Twice he had had snipe with interwoven feathers strapped on to the site of a fracture of one or other limb. The most interesting example was that of a snipe, both of whose legs he had unfortunately broken by a misdirect shot. He only recovered it on the following day, when he found that the poor creature had contrived to apply dressings and a sort of splint to both limbs. In carrying out this operation, some feathers had become entangled around the beak, and not being able to use its claws to get rid of them, it was almost dead from hunger when found. In a case recorded by M. Magnin, a snipe which was observed to fly away with a broken leg was subsequently found to have forced the fragments into a parallel position, and they were secured there by means of a strong band of feathers and moss intermingled. We always regarded Geneva as a sober and learned town !

One of the Sunday papers is in the habit of giving a paragraph headed "Drink and Death in London," under which it generally manages to give an account of two or three Saturday inquests on the victims of intemperance. Whether the readers are impressed by the obvious moral of such a paragraph we know not, but they certainly ought to be. Here is the evidence of one death : "The last three weeks she had drank heavily, taking no food, or undressed herself for days. On Wednesday, when he came home, he found her on the floor and he carried her to bed. She was fully dressed as usual; he saw nothing more of her until the following morning at seven o'clock, when he was going to his work, and he then found her under the table ; he again put her on the bed, and she died about twenty minutes afterwards. Dr. Hibbert said the cause of death was syncope following exhaustion produced by chronic alcoholism." In the case of another woman it was also stated that she would remain in her bed for days, speak to no one, and take no food ; in fact, do nothing but drink. The case of a man on whom an inquest was held the same time was thus described : "For years he had been of intemperate habits, taking no food. On Wednesday afternoon he suddenly fell down at the corner of Richmond-road, Westbourne-grove, and was taken to St. Mary's Hospital, where he died shortly after. Mr. T. Honey, house surgeon, said death was due to intemperate habits."

---

The Health Congress at Hastings has been well attended, and most of the papers read have been followed by lively discussion. Mr. Wilson Noble delivered an address on sanitary legislation, and was accorded a vote of thanks on the motion of Earl Fortescue. Papers were read by Charles Drysdale, M.D.; Hugh Alexander, chairman of the Association of Public Sanitary Inspectors of Great Britain ; Richard Greene, F.R.C.P. Edinburgh, superintendent of the county asylum, Northampton ; Shirley Murphy, hon. sec. Epidemiological Society ; and Sir Edwin Chadwick, the latter of whom advocated a yearly census and a new mode of keeping local accounts of human life rates. Major-General Webber, C.B., who delivered an important address on "Lighting of towns in relation to health," warmly advocating the superiority of the electric light, which system of lighting, he argued, must become general and be produced nearly as cheaply as gas in the near future. He specially commended the light on sanitary grounds. Professor Ayrton advocated gas for heating, and the electric current for lighting and ventilation, observing that if gas were generally used for heating it would do away with the dark clouds now hanging over great cities. At Thursday afternoon sitting the question of the disposal of the dead was discussed, a paper being contributed by Sir Spencer Wells, F.R.C.S., who began by giving an account of the objects and constitution of the Cremation Society. For fifteen years that society had been working and had overcome much prejudice, doubt, and fear. After dwelling on the advantages of cremation over ordinary burial, he said it was now very easy to ensure the cremation of a dead body, provided death from natural causes could be proved, and concluded by quoting from an address delivered on the subject by the late Bishop of Manchester. Mr. F. Seymour Haden and Mr. Keith D. Young also read papers. Dr. B. W. Richardson, president of the congress, said that hitherto he had opposed cremation vigorously on medical legal grounds. Lately, however, difficulties of this character had been removed, and in large towns like London he thought it almost impossible to do without cremation. Though he would prefer cremation for his own body, he believed that the earth-to-earth system could be carried out in a perfectly sanitary manner, and he would allow this system to go on side by side with cremation. Pleasant excursions were made by members of the congress, and the exhibition was largely patronised.

